# The next-generation sequencing reveals the complete mitochondrial genome of *Rhinogobius formosanus* (Perciformes: Gobiidae)

**DOI:** 10.1080/23802359.2020.1787261

**Published:** 2020-07-06

**Authors:** Chaojie Yang, Yan Chen, Zhi Chen, Guohao He, Ziliang Zhong, Wenbo Xue

**Affiliations:** College of Fisheries and Life Science, Hainan Tropical Ocean University, Sanya, China

**Keywords:** Mitochondrial genome, *Rhinogobius formosanus*, phylogenetic analysis

## Abstract

The complete mitochondrial genome of the *Rhinogobius formosanus* is presented in this study. In brief, it is 16,500 bp long and consists of 13 protein-coding genes, two rRNA genes, 22 tRNA genes, and a control region. The gene order and composition were similar to those of most other vertebrates. The nucleotide compositions of the heavy strand are 16.6% of G, 26.0% of T, 27.7% of A, and 29.8% of C. With the exception of the NADH dehydrogenase subunit 6 (ND6) and eight tRNA genes, all other mitochondrial genes are encoded on the heavy strand. The phylogenetic analysis by neighbour-joining (NJ) method showed that the *R. formosanus* has the closer relationship to *Rhinogobius leavelli* in the phylogenetic relationship.

*Rhinogobius formosanus* Oshima, 1919 is previously endemic to river systems of northern and northeastern Taiwan, China (Suzuki et al. 2012). However, it is now spreading to other suitable aquatic ecosystem by aquarium trade (Riede 2004). Several studies have been carried out regarding the morphology, systematic, migration, and ecology of this species (Oshima 1919; Chen and Shao 1996; Riede 2004; Suzuki et al. 2012). However, studies on the genetic diversity of *R. formosanus* have little been conducted yet. Assessments of genetic information are essential to develop strategies for the identification and management of fisheries resources. The next-generation sequencing (NGS) technologies, such as Illumine, allow considerable numbers of sequence data to be rapidly and efficiently characterized, which makes it particularly feasible for mitogenomes (Gilbert et al. 2007). Moreover, Illumine has been successfully used to assemble the mitogenomes of fish species (Cui et al. 2009). Therefore, we determined to sequence the complete mitochondrial genome of *R. formosanus* using the next-generation sequencing (NGS) techniques strategy in order to find DNA markers for the studies on the genetics of *R. formosanus*.

The specimens of *R. formosanus* were collected from the ornamental fish market of Sanya (18.11°N, 118.58°E), China during August 2019. All three examined specimens have been deposited in the College of Fisheries and Life Science, Hainan Tropical Ocean University, Sanya, China (Voucher number: HTOU-CFLS-0845 to HTOU-CFLS-0847). The HTOU-CFLS-0845 was used to extract total genomic DNA. The genomic DNA was extracted from dorsal-lateral muscles (30 mg) using Rapid Animal Genomic DNA Isolation Kit (Sangon Biotech Co., Ltd., Shanghai, CN). A genomic library was established followed by next-generation sequencing. Quality check for sequencing data was done by FastQC (Andrews 2010) and the fragments sequences were assembled and mapped using Spades v3.9.0 (Bankevich et al. 2012).

The final sequence has been deposited in GeneBank with accession number MT363639 (https://www.ncbi.nlm.nih.gov/nuccore/MT363639). The complete mitochondrial genome of *R. formosanus* (16,500 bp in length) consists of 13 protein-coding genes, 22 transfer RNA genes (tRNA), two ribosomal RNA genes (12S rRNA and 16S rRNA), and two non-coding control regions (control region and origin of light-strand replication). The arrangement of all genes is identical to that of most vertebrates (Wang et al. 2008; Chen 2013; Chiang et al. 2013). Most of the genes are encoded on the heavy strand (H-strand), except for the eight tRNA genes (-Gln, -Ala, -Asn, -Cys,-Tyr, -Ser, -Glu and -Pro) and one protein-coding gene (NADH dehydrogenase subunit 6, ND6). The overall nucleotide compositions of the heavy strand in descending order are 16.6% of G, 26.0% of T, 27.7% of A, and 29.8% of C, with a slight A + T-rich feature (53.7%). All the protein-coding genes begin with an ATG start codon except for COI started with GTG. Three types of stop codons revealed are TAA (COI, ATP8, ATP6, COIII, ND4L, ND5), TAG (ND1, ND2, ND3, ND6), and T (COII, ND4, Cytb). These features are common among vertebrate mitochondrial genome, and TAA is supposed to be appeared via posttranscriptional polyadenylation (Ojala et al. 1981). The longest one is ND5 gene (1839 bp) among protein-coding genes, whereas the shortest is ATPase 8 gene (165 bp). The two ribosomal RNA genes, 12S rRNA (951 bp) and 16S rRNA (1684 bp), are located between tRNA-Phe (GAA) and tRNA-Leu (TAA), and are separated by the tRNA-Val gene with the same situation found in other vertebrates. Most genes are either abutted or overlapped. The 22 tRNA genes vary from 69 to 77 bp in length. All these could be folded into the typical cloverleaf secondary structure except tRNA-Ser (AGY), and although numerous non-complementary and T-G base pairs exist in the stem regions. The control region was 842 bp in length, located between tRNA-Pro (TGG) and tRNA-Phe (GAA) gene. The nucleotide composition of control region was 30.17% of A, 21.85% of C, 16.63% of G, 31.35% of T.

To confirm the phylogenetic position of *R. formosanus* among genus Rhinogobius, a neighbour-joining (NJ) tree was reconstructed with the complete mtDNA sequences from five species. As shown in Figure 1, the *R. formosanus* has the closer relationship to *Rhinogobius leavelli*. The mitogenome information will be beneficial for future phylogenetic studies and specimen identification of Rhinogobius species.

**Figure 1.**
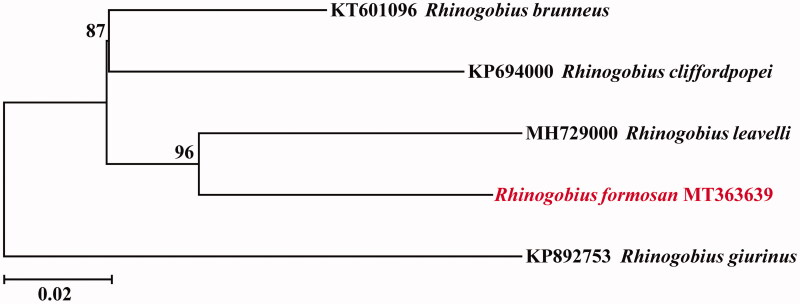
Phylogenetic relationships using NJ algorithm among same genus species. Numbers on each node are bootstrap values of 1000 replicates.

## Data Availability

The data that support the findings of this study are openly available in GenBank of NCBI at https://www.ncbi.nlm.nih.gov, reference number MT363639.

## References

[CIT0001] Andrews S. 2010. FastQC: a quality control tool for high throughput sequence data. [accessed 2018 Oct 4]. http://www.bioinformatics.babraham.ac.uk/projects/fastqc/.

[CIT0002] Bankevich A, Nurk S, Antipov D, Gurevich AA, Dvorkin M, Kulikov AS, Lesin VM, Nikolenko SI, Pham S, Prjibelski AD, et al. 2012. SPAdes: a new genome assembly algorithm and its applications to single-cell sequencing. J Comput Biol. 19(5):455–477. 22506599 10.1089/cmb.2012.0021 PMC3342519

[CIT0003] Chen IS. 2013. The complete mitochondrial genome of Chinese sucker *Myxocyprinus asiaticus* (Cypriniformes, Catostomidae). Mitochondrial DNA. 24(6):680–682. 23544832 10.3109/19401736.2013.773985

[CIT0004] Chen IS, Shao KT. 1996. A taxonomic review of the Gobiid fish genus *Rhinogobius* Gill, 1859, from Taiwan, with descriptions of three new species. Zool Stud. 35:200–214.

[CIT0005] Chiang TY, Chen IS, Lin HD, Chang WB, Ju YM. 2013. Complete mitochondrial genome of *Sicyopterus japonicus* (Perciformes, Gobiidae). Mitochondrial DNA. 24(3):191–193. 23316698 10.3109/19401736.2012.744980

[CIT0006] Cui Z, Liu Y, Li CP, You F, Chu HK. 2009. The complete mitochondrial genome of the large yellow croaker, *Larimichthys crocea* (Perciformes, Sciaenidae): unusual features of its control region and the phylogenetic position of the Sciaenidae. Gene. 432(1–2):33–43. 19100818 10.1016/j.gene.2008.11.024

[CIT0007] Gilbert MTP, Tomsho LP, Rendulic S, Packard M, Drautz DI, Sher A, Tikhonov A, Dalen L, Kuznetsova T, Kosintsev P, et al. 2007. Whole genome shot gun sequencing of mitochondria from ancient hair shafts. Science. 317(5846):1927–1930. 17901335 10.1126/science.1146971

[CIT0008] Ojala D, Montoya J, Attardi G. 1981. tRNA punctuation model of RNA processing in human mitochondria. Nature. 290(5806):470–474. 7219536 10.1038/290470a0

[CIT0009] Oshima M. 1919. Contributions to the study of the fresh water fishes of the island of Formosa. Ann Carnegie Mus. 12:269–272.

[CIT0010] Riede K. 2004. Global register of migratory species-from global to regional scales. Final Report of the R&D-Project. Bonn (Germany): Federal Agency for Nature Conservation. p. 329.

[CIT0011] Suzuki T, Chen IS, Senou H. 2012. A new species of *Rhinogobius* Gill, 1859 (Teleostei: Gobiidae) from the Bonin Islands, Japan. J Mar Sci Technol. 19:693–701.

[CIT0012] Wang C, Chen Q, Lu G, Xu J, Yang Q, Li S. 2008. Complete mitochondrial genome of the grass carp (Teleostei, Cyprinidae, Gobioninae). Gene. 424(1–2):96–101. 18706492 10.1016/j.gene.2008.07.011

